# Neuromyelitis optica is an HLA associated disease different from Multiple Sclerosis: a systematic review with meta-analysis

**DOI:** 10.1038/s41598-020-80535-3

**Published:** 2021-01-08

**Authors:** Marcos Papais Alvarenga, Luciana Ferreira do Carmo, Claudia Cristina Ferreira Vasconcelos, Marina Papais Alvarenga, Helcio Alvarenga-Filho, Cleonice Alves de Melo Bento, Carmen Lucia Antão Paiva, Laura Leyva-Fernández, Óscar Fernández, Regina Maria Papais-Alvarenga

**Affiliations:** 1 Programa de Pós-Graduação em Neurologia, Universidade Federal do Estado do Rio de Janeiro (UNIRIO), Rua Mariz e Barros 775, Rio de Janeiro, RJ 20270-004 Brazil; 2 Departamento de Neurologia, Hospital Federal da Lagoa, Rua Jardim Botânico 501, Rio de Janeiro, RJ 22470-050 Brazil; 3 Universidade Estácio de Sá (UNESA), Avenida Ayrton Senna, 2800, Barra da Tijuca, Rio de Janeiro, RJ 22775-003 Brazil; 4 Instituto de Investigación Biomédica de Málaga-IBIMA, UGCNeurociencias, Hospital Regional Universitario de Málaga, Avenida de Carlos Haya sn, 29010 Málaga, Spain; 5 Red Temática de Investigación Cooperativa: Red Española de Esclerosis Multiple REEM (RD 16/0015/0010), Barcelona, Spain; 6 Instituto de Investigación Biomédica de Málaga-IBIMA, Hospital Regional Universitario de Málaga, Avenida de Carlos Haya sn, 29010 Málaga, Spain

**Keywords:** Neurological disorders, Genetic association study, Immunogenetics, Neuroimmunology, Immunological disorders, Genetics, Molecular biology, Biomarkers

## Abstract

Neuromyelitis Optica and Multiple Sclerosis are idiopathic inflammatory demyelinating diseases of the central nervous system that currently are considered distinct autoimmune diseases, so differences in genetic susceptibility would be expected. This study aimed to investigate the HLA association with Neuromyelitis Optica by a systematic review with meta-analysis. The STROBE instrument guided research paper assessments. Thirteen papers published between 2009 and 2020 were eligible. 568 Neuromyelitis Optica patients, 41.4% Asians, 32.4% Latin Americans and 26.2% Europeans were analyzed. Only alleles of the *DRB1* locus were genotyped in all studies. Neuromyelitis Optica patients have 2.46 more chances of having the DRB1*03 allelic group than controls. Ethnicity can influence genetic susceptibility. The main HLA association with Neuromyelitis Optica was the DRB1*03:01 allele in Western populations and with the DPB1*05:01 allele in Asia. Differences in the Multiple Sclerosis and Neuromyelitis Optica genetic susceptibility was confirmed in Afro descendants. The DRB1*03 allelic group associated with Neuromyelitis Optica has also been described in other systemic autoimmune diseases.

## Introduction

Multiple Sclerosis (MS) and Neuromyelitis optica (NMO) are inflammatory and neurodegenerative diseases of the central nervous system, that preferentially affect young woman causing neurological dysfunctions and disability^1^.

MS is the most frequent Idiopathic Inflammatory Demyelinating Diseases (IIDD), disseminated in time and space and a typical relapsing remitting clinical course. It has a peculiar geographical distribution, with a high prevalence in Caucasian of the Northern Hemisphere, and a very low prevalence in populations living in tropical regions^2,3^. NMO is a rare disease that occurs more frequently in Asians and Afro-descendants and is characterized, in most cases, by selective but not exclusive involvement of the optic nerve and spinal cord, also evolving with a relapsing remitting clinical course^4^.

It was not until the 90th decade that MS and NMO were recognized as distinct immune mediated diseases; NMO differs from MS in its demographic distribution, resonance magnetic images, morbidity, and pathogenesis^5–7^. Identifying a serum immunoglobulin G autoantibody class, the NMO-IgG, with high specificity for NMO, and not found in MS, strengthened the difference between these immune-mediated diseases. It has been shown that the NMO-IgG selectively binds to aquaporin-4 (AQP4), a water channel consisting of a transmembrane protein located at the terminal feet of the astrocytes in the blood–brain barrier. AQP4 is involved with the function and integrity of this barrier^8,9^.

NMO spectrum disorders (NMOSD) was coined to include all rare CNS syndromes where the NMO-IgG was found at different frequencies. The NMOSD comprises NMO and high-risk syndromes (HR-NMO) as bilateral or recurrent optic neuritis (BRON), longitudinally extensive transverse myelitis (LETM), ON or LETM with brainstem/encephalopathy or associated with other systemic autoimmune diseases and also Asian optic spinal Multiple Sclerosis (OSMS)^10,11^. Studies in Japan applying new laboratory techniques, identified the AQP4-IgG only in OSMS with longitudinally extensive spinal cord lesions (LESCLS), and since them, those cases are considered similar to NMO^12,13^. A new classification for NMOSD proposed by an international panel stratified the cases by the AQP4-Ab status (positive/unknown or negative) and considered OSMS with LESCLS similar to NMO^14^.

A subset of NMO patients that were negative for AQP4-IgG showed positivity for antibodies against the myelin oligodendrocyte glycoprotein (MOG-IgG)^15^. Currently, NMO is defined as an astrocytopathy mediated by AQP4-IgG. MOG-IgG positive cases are related to a spectrum of demyelinating syndromes of the CNS denominated MOGADs^1,16^.

Although the etiology of the CNS's immune-mediated diseases remains unclear, the influence of environmental and genetic factors in the pathogenesis of MS is well recognized. The knowledge about the genetic bases of MS has been acquired in the last 40 years. The discovery of association between human leukocyte antigen (HLA) DRB1*15 allelic variants and MS, the identification of MS cluster in families, and the higher concordance rate in monozygotic twins (20–30%) than dizygotic twins (2–5%), and the high incidence in some ancestral groups irrespective of the geographic location, provided shreds of evidence to classify MS as a complex genetic disease, with moderate heritability, polygenic inheritance, and multifaceted gene-environment interaction^17^.

Considering that MS and NMO are distinct CNS immune-mediated diseases, differences in genetic susceptibility would be expected. Few data about the genetics of NMO are available. Familial aggregation is uncommon^18,19^, the occurrence in twins is exceptional^20^, but the major distribution of the disease in Asian and African descendants^4^ suggests a genetic influence.

In Japan, differences in genetic susceptibility between Asian and Western-type MS were described in the 1990s^21,22^. In Western countries, a possible difference between the HLA allelic profile of MS and NMO was made in a case study of six Canadian aborigines initially diagnosed with MS; however, further necropsy demonstrated NMO characteristics. Besides, one pacient had *HLA* DRB1*15, and none of the patients had the *HLA DQB1* type that were previously reported with hight frequency among MS patients ^23^.

The first case–control study investigating the HLA Class I and Class II *DR*, *DQ*, *DP* alleles in French Caucasians with NMO, MS, and Healthy Controls (HC) was published in 2009. An association of HLA Class II DRB1*03 allelic group with NMO was described, and the analysis of the distribution of HLA-*DRB1* showed significant differences between the NMO and the MS groups^24^.

The main objective of this systematic review was to analyze studies investigating the HLA association with NMO. Another goal was to verify possible differences between the genetic susceptibility of NMO and MS which would favour the distinction between the CNS's immune demyelinating diseases.

## Results

### Eligible studies

**Figure 1:**
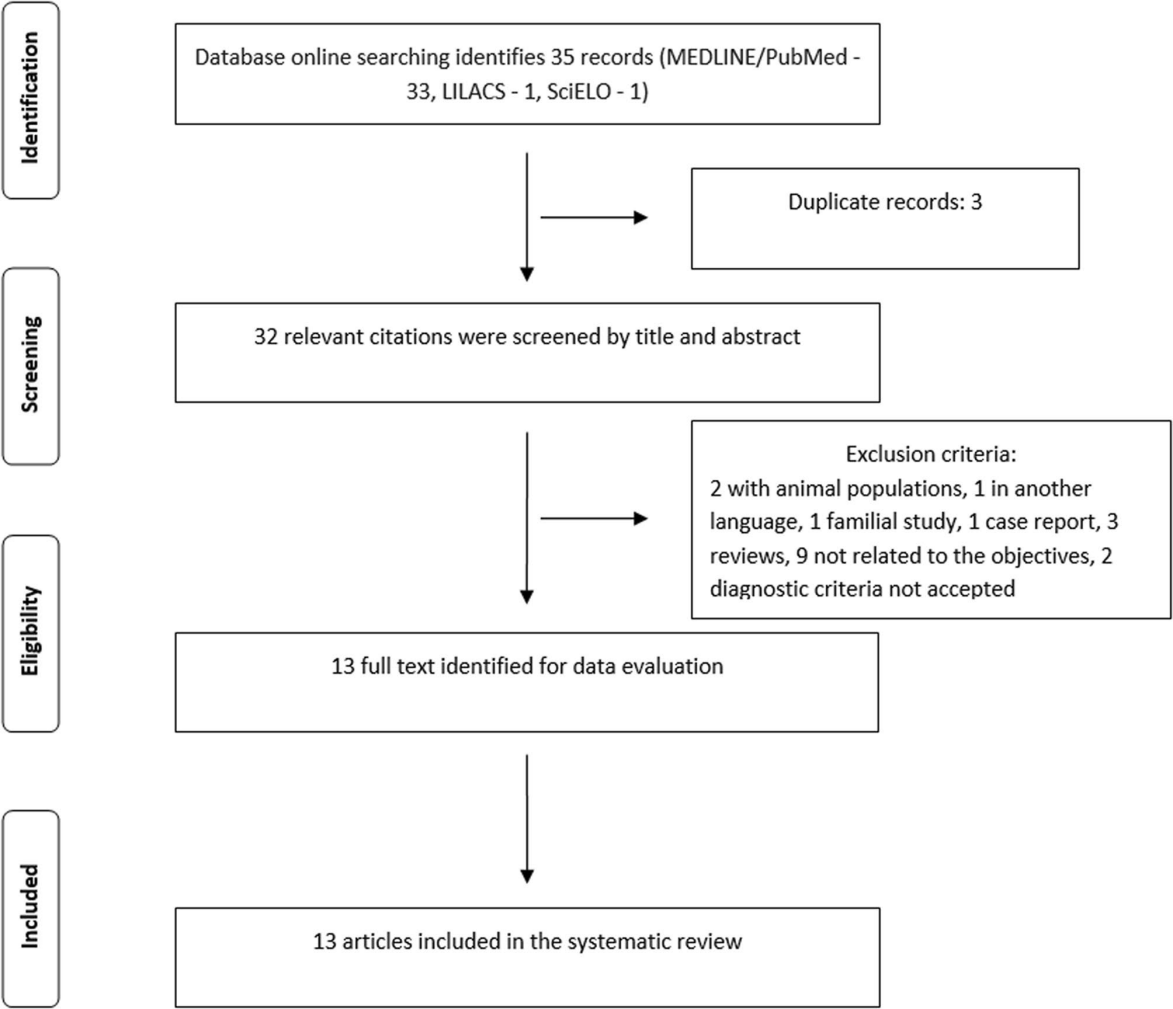
Study identification flowchart. Study identification flowchart following the PRISMA statement^60^. *MEDLINE* Medical Literature Analysis and Retrieval System Online, *LILACS* Scientific and Technical Literature of Latin America and the Caribbean, *SciELO* Scientific Electronic Library Online.

The search strategy determined in the methodology and executed until March 31, 2020, identified 35 articles in the LILACS, SciELO, and PubMed databases. Papers found in more than one database were considered only once, thus totaling 32 articles. The PRISMA Statement flowchart of information is shown in Fig. 1. After applying the inclusion and exclusion criteria, 13 articles were selected for this review, as shown in Table 1^24–36^. The eligible studies analyzed European Caucasians (France^24^, Spain^27^, Denmark^29^ and Netherland^36^), Mexico mestizos^34^, Afro Caribbean^26^, Afro Brazilians (Ribeirão Preto^25^, Rio de Janeiro^33^), White Brazilians (South Region^35^) and Asians (South China^28^, South Japan^30^, India^31^ and Israel^32^). There was agreement among the evaluators about the selection of articles.

**Table 1:** Articles included in the systematic review.

Study authors	Year	Journal	Studies location and ethnicity	Studies
Zéphir et al.^24^	2009	Multiple Sclerosis Journal	France (Caucasian)	Is neuromyelitis optica associated with human leukocyte antigen?
Brum et al.^25^	2010	Multiple Sclerosis Journal	Brazil, SP, Ribeirão Preto (Mulatto)	*HLA-DRB* association in neuromyelitis optica is different from that observed in multiple sclerosis
Deschamps et al.^26^	2011	Multiple Sclerosis Journal	French West Indies (Afro-Caribbean)	Different HLA class II (*DRB1* and *DQB1*) alleles determine either susceptibility or resistance to NMO and multiple sclerosis among the French Afro-Caribbean population
Blanco et al.^27^	2011	Revista de Neurología	Spain (Caucasian)	*HLA-DRB1* typing in Caucasians patients with neuromyelitis optica
Wang et al.^28^	2011	Journal of Neuroimmunology	China (Southern Han)	The HLA-DPB1*05:01 is associated with susceptibility to anti-aquaporin-4 antibodies positive neuromyelitis optica in Southern Han Chinese
Asgari et al.^29^	2012	Multiple Sclerosis Journal	Denmark (Caucasian)	*HLA, PTPN22* and *PD-1* associations as markers of autoimmunity in neuromyelitis optica
Yoshimura et al.^30^	2013	Journal of neurology, neurosurgery and Psychiatry	South Japan (Asian)	Distinct genetic and infectious profiles in Japanese neuromyelitis optica patients according to anti-aquaporin 4 antibody status
Pandit et al.^31^	2015	Multiple Sclerosis Journal	South India (Indian)	Human leukocyte antigen association with neuromyelitis optica in a south Indian population
Brill et al.^32^	2016	Journal of Neuroimmunology	Israel (ArabMuslim)	Increased occurrence of anti-AQP4 seropositivity and unique HLA Class II associations with neuromyelitis optica (NMO), among Muslim Arabs in Israel
Alvarenga et al.^33^	2017	Journal of Neuroimmunology	Brazil—RJ (70% Afro-descendant)	The HLA-DRB1*03:01 allele is associated with NMO regardless of the NMO-IgG status in Brazilian patients from Rio de Janeiro
Alonso et al.^34^	2018	Central Nervous System Agents in Medicinal Chemistry	Mexico (Mestizo)	Neuromyelitis Optica (NMO IgG+) and Genetic Susceptibility, Potential Ethnic Influences
Kay et al.^35^	2019	Arquivos de Neuro-Psiquiatria	South Brazil (80% white)	HLA-alleles class I and II associated with genetic susceptibility to neuromyelitis optica in Brazilian patients
Bruijstens et al.^36^	2020	Neurology Neuroimmunology & Neuroinflammation	Netherlands (Caucasian)	HLA association in MOG-IgG- and AQP4-IgG-related disorders of the CNS in the Dutch population

### Participants

Table 2 indicates the participants’ characteristics and the description of the genotyped *HLA DR*/*DQ* alleles. A total of 568 NMO patients were genotyped: 41.4% Asians, 32.4% Latin Americans and 26.2% European Caucasians. 502 cases full filled the NMO diagnostic criteria^10^, 54 had high-risk NMO syndromes, and 12 were classified as NMOSD^11^. The NMO-IgG was tested in 314 patients in seven studies, and 164 (52.2%) tested positive for this antibody. Other six studies selected 225 cases, which also tested positive for the antibody. Overall, 389 (68.5%) of the NMO patients were positive for NMO-IgG.

**Table 2:** Subjects collected data and genotyped HLA Class II alleles.

Studies	Studies subjects			NMO/NMOSD	Genotyped HLA alleles at the *DR* and *DQ* loci			
	NMO	MS	Controls	Methodology and frequency of NMO-IgG/AQP-4 Ab positivity	Analysis method resolution	*DRB1* locus	*DQA1* locus	*DQB1* locus
France^24^	45 (39 NMO, 2RON, 4 LETM)	161	310	IIF 24/45–53.3% (18/39 NMO, 6/6 NMOSD tested)	High resolution	9 alleles *01, *03, *04, *07, *8–9-10, *11–12, *13–14, *15, *16	7 alleles *01:01, *01:02, *01:03–04, *02:01, *03:01, *04:01, *05:01	10 alleles *02:01, *03:01, *03:02, *03:03–04, *04:02, *05:01, *05:02–03, *06:02, *06:01–03, *06:04
Brazil (SP)^25^	27 (17 NMO, 2 RON, 8 LETM)	29	28	IIF 100%	Low resolution	13 alleles *01, *03, *04, *07, *08, *09, *10, *11, *12, *13, *14, *15, *16, *DRB3*, *DRB4*, *DRB5*		
French West Indies^26^	42	163	150	Not specifiedtechnique 13/29–44.8% (29/42 tested)	Medium resolution	15 alleles *01, *03, *04, *05, *06, *07, *08, *09, *10, *11, *12, *13, *14, *15, *16		10 alleles *01, *02, *03, *04, *05, *06
Spain^27^	22	228	225	IIF and CBA 16/22–72.7%	Medium resolution	13 alleles *01, *03, *04, *07, *08, *09, *10, *11, *12, *13, *14, *15, *16		
South China^28^	30	53	93	IFF 100%	High resolution	26 alleles *01:01, *03:01, *04:01, *04:03, *04:04, *04:05, *04:06, *04:10, *07:01, *08:02, *08:03, *09:01, *10:01, *11:01, *11:06, *12:01, *12:02, *13:01, *13:02, *14:02, *14:03, *14:05, *14:06, *14:54, *15:01, *15:02, *16:01, *16:02		
Denmark^29^	41 (35 NMO, 5 BRON, 1 LETM)	42	200	IIF and CBA 25/41–61% (19/35 NMO, 6/6 NMOSD tested)	Medium and high Resolution	13 alleles *01, *03, *04, *07, *08, *09, *10, *11, *12, *13, *14, *15, *16	6 alleles *01, *02:01, *03, *04:01, *04:04, *05	5 alleles *02, *03, *04:02, *05, *06
Japan^30^	77 (65 NMO) 12 NMOSD)		367	CBA 100 %	High resolution	28 alleles *01:01, *03:01, *04:01, *04:03, *04:04, *04:05, *04:06, *07:01, *08:01, *08:02, *08:03, *09:01, *10:01, *11:01, *12:01, *12:02, *13:01, *13:02, *13:12, *14:04, *14:05, *14;18, *14:54, *15:01, *15:02, *16:02		
India^31^	93 (61 NMO, 20 RON, 11 LETM, 1 RTD)	300	300	CBA 44/93–47.3%	Low and high resolution	10 alleles *01, *03, *04, *07, *10, *12, *13, *14, *15:01, *15:02	^a^	^a^
Israel^32^	35		74	ELISA and CBA 17/35–48.57%	Low and High resolution	23 alleles *01:01, *03:01, *04:01, *04:02, *04:03, *04:04, *04:05, *04:06, *07:01, *08, *09:01, *10:01, *11, *12:01,*13:01, *13:02, *13:03, *13:05, *14, *15:01, *15:02, *16:01, *16:02		19 alleles *02:01, *02:02, *02:04, *03:01, *03:02, *03:03, *03:04, *03:05, *03:10, *04:02, *05:01, *05:02, *05:03, *06:01, *06:02, *06:03, *06:04, *06:09, *06:10
Brazil (RJ)^33^	65	94	100	IIF 25/49–51% (49/65 NMO tested)	High resolution	30 alleles *01:01, *01:02, *03:01, *04:01, *04:02, *04:03, *04:04, *04:05, *07:01, *08:01, *08:02, *08:03, *08:04, *09:01, *10:01, *11:01, *11:02, *11:03, *11:04, *12:01, *13:01, *13:02, *13:03, *14:01, *14:02, *15:01, *15:02, *15:03, *16:01, *16:02	12 alleles *01:01, *01:02, *01:03, *01:04, *01:05, *02:01, *03:01, *03:02, *03:03, *04:01, *05:01/3/5, *06:01	16 alleles *02:01, *02:02, *03:01, *03:02, *03:03, *03:04, *04:02, *05:01, *05:02, *05:03, *06:01, *06:02, *06:03, *06:04, *06:08, *06:09
Mexico^34^	35		99	Not specifiedtechnique 100%	Medium resolution	13 alleles *01, *03, *04, *07, *08, *09, *10, *11, *12, *13, *14, *15, *16		
South Brazil^35^	15		252	IIF 100%	High resolution	7 alleles *03:01, *04:05, *10:01, *16:02, *01:01, *07:01, *03:02		6 alleles *05:01, *02:01, *03:01, *03:02, *03:19, *04:02
Netherlands^36^	41		5.514	CBA 100%	Medium resolution	17 alleles *01, *03, *04, *07, *08, *09, *10, *11, *12, *13, *14, *15, *16, *DR18*, *DRB3*, *DRB4*, *DRB5*		7 alleles *2, *4, *5, *6, *DQ7*, *DQ8*, *DQ9*

*NMO* NeuromyelitisOptica, *NMOSD* NeuromyelitisOpticaSpectrum Disorders, *MS* Multiple Sclerosis, *IgG* Immunoglobulin G, *AQP-4* Aquaporin-4, *HLA* Human LeukocyteAntigen, *RON* RecurrentOptic Neuritis, *BRON* Bilateral RecurrentOptic Neuritis, *LETM* Longitudinal ExtensiveTransverseMyelitis, *SP* São Paulo, *RJ* Rio de Janeiro, *IIF* indirectimmunofluorescenceassay, *CBA* cell-based assay, *ELISA* enzyme-linkedimmunosorbentassay.

^a^Data not described.

HC groups, composed of persons showing no demyelinating disease, varied from 28 to 5514 participants from the same geographic region. The susceptibility for MS was analyzed in eight elegible studies that described the frequency and association of the *HLA DRB1* alleles, for comparison, among the MS groups ranging from 29 to 300 MS patients^24–27,29,31,33^. One study also analyzed the HLA association with MOG-IgG disorders and NMO for comparison^36^.

The number of alleles genotyped in the *DRB1* locus varied from 7 to 30, 6–12 in *DQA1* locus, and 5–19 in *DQB1* locus. Five studies used a high-resolution technique for typing HLA alleles in all studied loci.

### HLA association with NMO

The case–control studies’ results comparing the allelic frequency of the DRB1*03 allele group in NMO with local controls are shown in Table 3.

**Table 3:** Case controls studies that investigated the association of HLA DRB1*03 allelic group and DRB1*03:01 allele in NMO groups.

Studies	*HLA DRB1*	DRB1*03/*03:01 NMO vs Controls					DRB1*03/*03:01 NMO subgroups vs Controls							
		Allelic frequency (2n) or Phenotypic frequency (n) %		*OR*	CI	*p* value	Allelic frequency (2n) or Phenotypic frequency (n) %		AQP-4 Ab (+) vs Controls			AQP-4 Ab (−) vs Controls		
		NMO	Controls				AQP-4 Ab ( +)	AQP-4 Ab (−)	*OR*	CI	*p* value	*OR*	CI	*p* value
France^24^ (2n)	*03	22	11	2.32	1.32–4.04	*p*^*cB*^ 0.02	27	16	3.08	1.52–6.27	*p*^*cF*^ 0.01	1.56	0.67–3.63	*p*^*cF*^ NS
Brazil (SP)^25^ (2n)	*03	24.1	8.9	3.23	1.07–9.82	*p*^*cF*^ 0.04								
French West Indies^26^ (2n)	*03	26.2	13	2.4	1.31–4.28	*p*^*cB*^ 0.045	26.9	25	2.46	0.82–6.61	*p*^*cF*^ NS	2.22	0.81–5.58	*p*^*cF*^ NS
Spain^27^ (2n)	*03	20.4	10.9	2.10	0.95–4.64	*p*^*cB*^ NS	^b^	^b^	^b^	^b^	^b^	^b^	^b^	^b^
South China^28^ (n)	*03:01	23.3	13.2	^b^	Not specified	*p*^*cB*^ NS								
Denmark^29^ (2n)	*03	21	15	1.48	0.81–2.7	*p*^*cB*^ NS	24	16	1.79	0.89–3.62	*p*^*cB*^ NS	1.05	0.39–2.8	*p*^*cB*^ NS
Japan^30^ (n)	*03:01	2.6	0.5	^b^	Not specified	*p*^*cB*^ NS								
India^31^ (2n)^a^	*03	11	2	5.69	2.39–13.5	*p*^*cB*^ 0.00009	13	^b^	9.23	2.62–32.46	*p*^*cB*^ 0.009	^b^	^b^	^b^
Israel^32^ (2n)	*03:01	10	10.1	^b^	Not specified	*p*^*cB*^ NS	8.82	9.38	^b^	^b^	*p*^*cB*^ NS	^b^	^b^	*p*^*cB*^ NS
Brazil (RJ)^33^ (n)	*03:01	41.5	22	2.52	1.27–4.99	*p*^*cF*^ 0.007	44	50	2.79	1.11–6.99	*p*^*cF*^ 0.026	3.54	1.4–8.98	*p*^*cF*^ 0.006
Mexico^34^ (2n)	*03	14	5	2.8	1.05–7.6	*p*^*cY*^ 0.03								
South Brazil^35^ (2n)	*03:01	16.7	56	3.4	1.21–9.55	*p*^*cB*^ NS								
Netherlands^36^ (n)	*03	51.2	27.6	2.75	1.5–5.04	*p*^*cS*^0.02								

NMO, Neuromyelitis Optica; IgG, Immunoglobulin G; AQP-4, Aquaporin-4; *OR*, *Odds Ratio*; CI, Confidence Interval; *p*^*cF*^, *p* corrected by Fisher’s exact test; *p*^*cB*^, *p* corrected by Bonferroni method; *p*^*cY*^, *p* corrected by Yates method; *p*^*cS*^corrected by Sidak method; SP, São Paulo; RJ, Rio de Janeiro.

^a^After stratification for AQP4 positivity no significant differences were observed between NMO subgroups and controls.

^b^Data not described.

In Europe, the association of DRB1*03 allele group in NMO was found in France^24^ (NMO-22.02% vs controls-11.0%, *p*^*cS*^ = 0.02) and in the Netherlands^36^ (NMO-51.2% vs controls-27.6%, *p*^*cS*^ = 0.02).

In Latin America, DRB1*03 allelic group were associated with NMO in Caribbean Islands^26^ (NMO-26.2% vs controls-13%, *p*^*cB*^ = 0.045), in Ribeirão Preto^25^ city (São Paulo, Brazil) (NMO—24.1% vs controls 8.9%, *p*^*cF*^ = 0.0401), in Mexico^34^ (NMO vs 14% vs controls 5%, *p* = 0.03) and in Rio de Janeiro^33^ (Brazil) (NMO—41.5% vs controls 22.2%, *p*^*cF*^ = 0.007).

In Asia, an association of the DRB1*03 allelic group with NMO was found in India^31^ (NMO 11% vs controls 2%, *p* = 0.00009).

**Figure 2:**
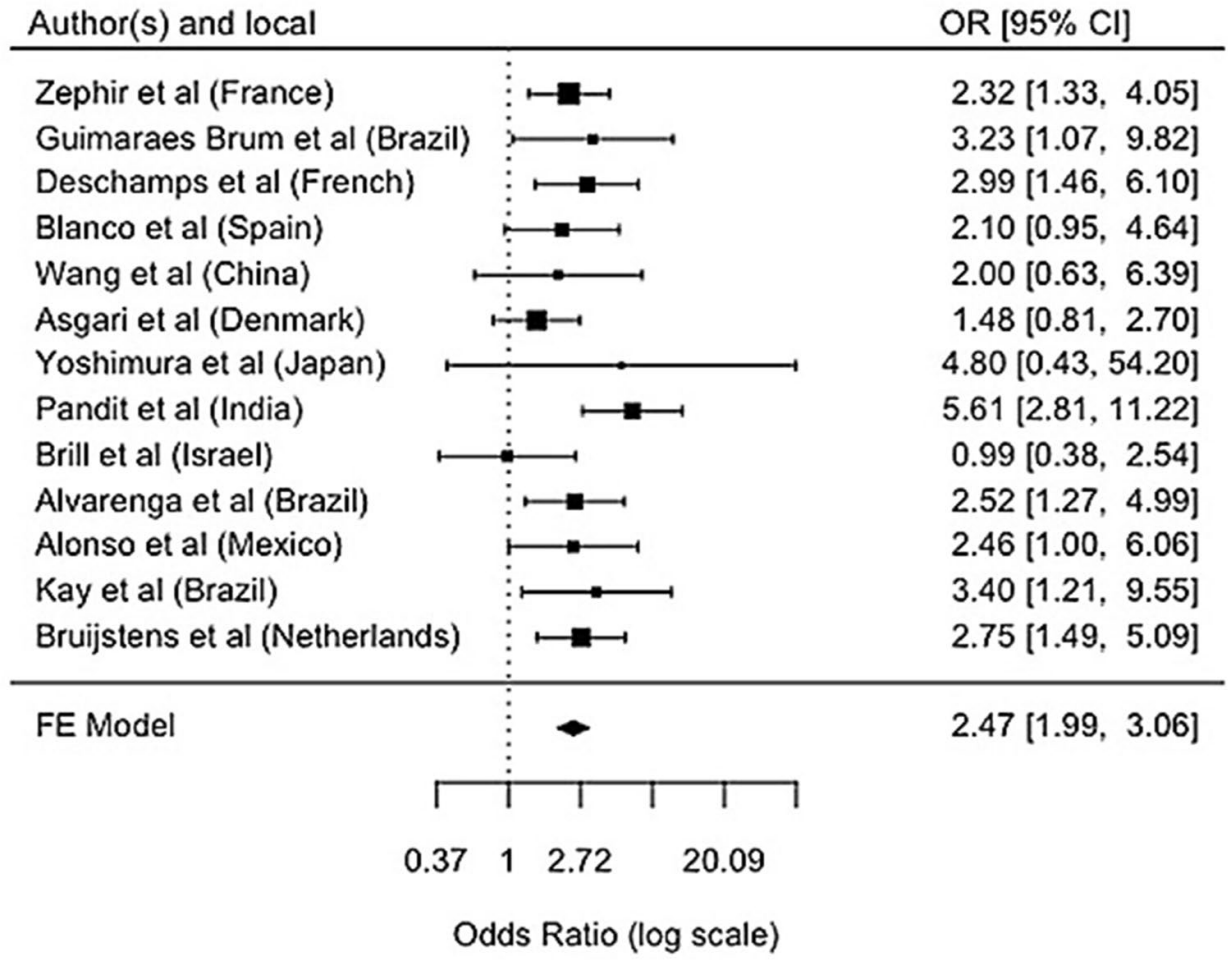
Meta-analysis: association of DRB1*03 allelic group with NMO. Comparison of DRB1*03 allele group association using meta-analysis based on the *OR* and the confidence interval (95% CI) described in the thirteen studies. The forest plot shows the summary measure of *OR* equal to 2.46 (95% CI 2.01–3.01). That is, patients with neuromyelitis optica are 2.46 times more likely to have the DRB1*03 allele group than controls. In the West, studies are not heterogeneous (I^2^ = 0.00%; *p* = 0.92), with the measure of *OR* equal to 2.38 (95% CI 1.90–2.97), but in Asia the result of the meta-analysis showed a heterogeneity of 67% (I^2^ = 66.91%; *p* = 0.02).

A meta-analysis with the results of the thirteen studies that investigated the association of the DRB1*03:01 allele with NMO is summarized at the forest plot (Fig. 2), indicating that patients with NMO are 2.46 times more likely to have the DRB1*03 allele group than controls (95% CI 2.01—3.01).

Table 4 describes the results of case controls studies showing the association of the HLA class II alleles (others than DRB1*03 allele group) and HLA class I alleles.

**Table 4:** Case control studies showing association with NMO of HLA class II alleles (others than DRB1*03allele group) and HLA class I alleles.

Studies	Class II alleles (*DR-DQ)* NMO vs Controls						Class I (*A, B, C*) and Class II alleles (*DP*) NMO vs Controls					
	*HLA*	Allelic frequency (2n) or phenotypic frequency (n) %		*OR*	CI	*p* value	HLA	Allelic frequency (2n) or phenotypic frequency (n) %		*OR*	CI	*p* value
		NMO	Controls					NMO	Controls			
South China^28^ (n)	DRB1*16:02	26.67	11.83	3.491	1.024–11.896	*p*^*cB*^ 0.038	DPB1*05:01	90.0	55.91	4.629	1.235–17.350	*p*^*cB*^ 0.018
Denmark^29^ (2n)	DQB1*04:02	9.0	3.0	3.64	1.34–9.87	*p*^*cB*^ 0.035						
Japan^30^ (n)	DRB1*16:02	6.9	0.8	8.988	2.344–34.468	*p*^*cB*^ 0.0223	DPB1*05:01	85.7	65.4	3.175	1.619–6.227	*p*^*cB*^ 0.0074
Brazil (RJ)^33^ (n)	DRB1*01:02	6.2	0	1.07	1.001–1.13	*p*^*cB*^ 0.023		44	50	2.79	1.11–6.99	*p*^*cF*^ 0.026
	DQA1*01:05	10.8	1	11.95	1.43–99.56	*p*^*cF*^ 0.007						
	DQB1*02:01	41.5	24	2.25	1.15–4.41	*p*^*cF*^ 0.017						
Mexico^34^ (2n)	DRB1*10	7.1	0.5	15.01	1.6–349.1	*p*^*cY*^ 0.005						
South Brazil^35^ (2n)	DRB1*04:05	10.0	0.4	27.89	4.47–173.97	*p*^*cB*^ 0.0016						
	DRB1*16:02	10.0	0.8	13.89	2.96–65.19	*p*^*cB*^ 0.0085						
Netherlands^36^ (n)							A*01	61.9	33.7	3.16	1.707–5.863	*p*^*cS*^ 0.0045
							B*08	61.9	25.6	4.66	2.513–8.643	*p*^*cS*^ 0.0000

NMO, Neuromyelitis Optica; *OR*, *Odds Ratio*; CI, Confidence Interval; *p*^*cF*^, *p* corrected by Fisher’s exact test; *p*^*cB*^, *p* corrected by Bonferroni method; *p*^*cY*^, *p* corrected by Yates method; *p*^*cS*^, corrected by Sidak method; SP, São Paulo; RJ, Rio de Janeiro.

The DPB1*05:01 allele was associated with NMO in China^28^ (NMO—90.0% vs controls—55.61%, *p*^*cB*^ = 0.018) and in Japan^30^ (NMO—85.7% vs controls—65.4%, *p* = 0.0074).

Although with low allele frequency, other HLA class I and II alleles were also associated with NMO as shown in Table 4. Most of these alleles were identified at the *DRB1* locus: DRB1*16:02 (China^28^, Japan^30^ and South Brazil^35^), DRB1*01:02 (Rio de Janeiro^33^), DRB1*10 (Mexico^34^), DRB1*04:05 (South Brazil^35^).

The association of class I HLA A*01 and B*08 with the NMO has only been described in Caucasians from the Netherlands^36^.

### HLA susceptibility in other CNS immune mediated diseases

Eight of the 13 case-controls studies elegible for this review also investigated the frequency of *DRB1* alleles in MS patients and Controls. An association with the HLA DRB1*15 allele group was found in Caucasians from France^24^ and Denmark^29^, Latin Americans from the Caribbean^26^, Brazil (SP^25^ and RJ^33^), and Asians from India^31^ showed at Table 5.

**Table 5:** Susceptibility in MS and comparison of NMO and MS groups regarding frequency of DRB1*15 and DRB1*03 allelic groups.

Studies	DRB1*15 MS vs controls					DRB1*03/*03:01 and DRB1*15 NMO vs MS					
	Allelic frequency (2n) %		*OR*	CI	*p* value	*DRB1*	Allelic frequency (2n) %		*OR*	CI	*p* value
	MS	Controls					NMO	MS			
France^24^	27	12	2.79	2.01–3.89	*p*^*cB*^ < 0.0008	*03	22	13	1.85	1.03–3.55	*p*^*cF*^ NS
						*15	19	27	0.57	0.32–1.01	*p*^*cF*^ NS
Brazil (SP)^25^	37.9	12.5	4.28	1.65–11.10	*p*^*cF*^ 0.0024	*03	24.07	8.62	3.23	1.07–9.82	*p*^*cF*^ 0.0254
						*15	3.7	37.9	15.89	3.51–71.85	*p*^*cF*^ 0.0001
French West Indies^26^	24.8	13	2.21	1.45–3.36	*p*^*cB*^ < 0.0015	*03	26.2	16.5	1.79	1.02–3.16	*p*^*cF*^ NS
						*15	8.3	24.8	0.27	0.12–0.61	*p*^*cF*^ 0.015
Spain^27^	18.6	12.5	1.61	1.12–2.32	*p*^*cB*^ NS	*03	20.4	13.4	1.60	0.74–3.50	*p*^*cB*^ NS
						*15	6.8	18.6	0.32	0.10–1.06	*p*^*cB*^ NS
China	22.6	21.5	^a^	^a^	*p*^*cB*^ NS	*03:01	23.3	13,2	^a^	^a^	*p*^*cB*^ NS
						*15:01	33.3	22.6	^a^	^a^	*p*^*cB*^ NS
Denmark^29^	35	17	2.61	1.56–4.41	*p*^*cB*^ 0.0027	*03	21	13	1.74	0.76–3.98	*p*^*cB*^ NS
						*15	30	35	0.80	0.42–1.54	*p*^*cB*^ NS
India^31^	21	13	1.62	1.01–2.67	*p*^*cB*^ 0.003	*03	12	6	0.46	0.21–1.04	*p*^*cB*^NS (< 0.01)
						*15:01	9	21	2.21	1.01–4.83	*p*^*cB*^ < 0.001
Brazil (RJ)^33^	15.4	4.5	4.397	1.88–10.31	*p*^*cF*^ 0.001	*03:01	20.8	6.4	4.43	2.06–9.52	*p*^*cF*^ < 0.001
						*15:01	2.3	15.4	0.13	0.04–0.44	*p*^*cF*^ < 0.001

MS, Multiple Sclerosis; NMO, Neuromyelitis Optica; *OR*, *Odds Ratio*; CI, Confidence Interval; *p*^*cF*^, *p* corrected by Fisher’s exact test; *p*^*cB*^, *p* corrected by Bonferroni method; NS, not significant.

^a^Data not described.

Only one study investigated the genetic susceptibility of MOGAD^36^ in Dutch patients. No association was found with HLA alleles class I or class II.

### Comparison between the NMO genetic susceptibility versus MS

**Figure 3:**
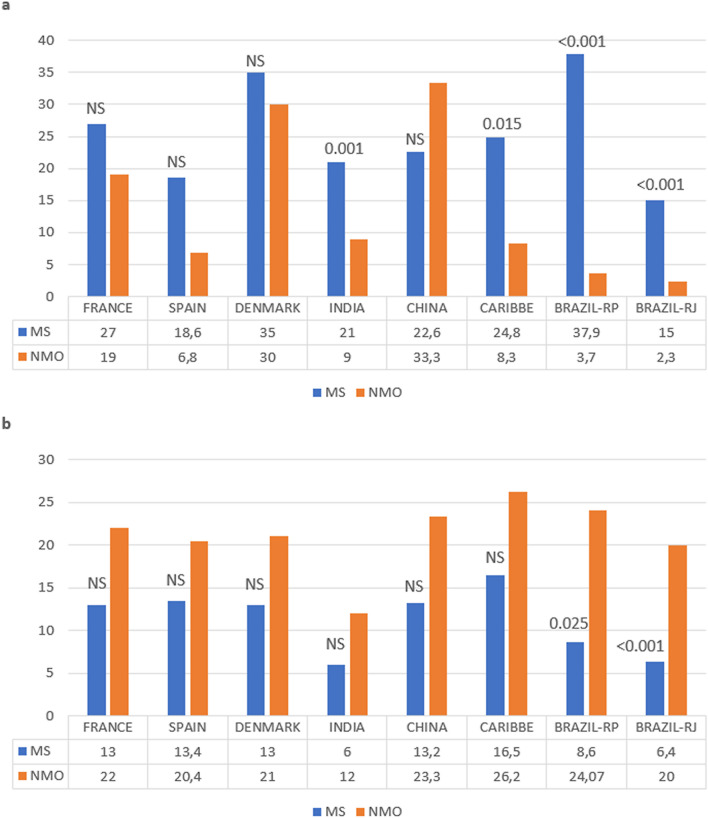
Distribution of the *HLA DRB1* alleles associated to NMO and MS in NMO and MS groups. (**a**) Comparison of the frequency of DRB1*15 allele group in the NMO and MS groups. (**b**) Comparison of the frequency of DRB1*03 allele group in the NMO and MS groups. Caucasians do not differ in terms of the *DRB1* allelic profile. Brazilian populations with strong African ancestry (Ribeirão Preto (RP) and Rio de Janeiro (RJ) had different distribution of *DRB1* alleles in NMO and MS groups. The significance (*p*) of each comparison is shown in the figure. *NS* not significant.

The frequency of the *DRB1* alleles associated with MS or NMO was compared in eight populations as shown at Table 5 and illustrated in Fig. 3. Two studies showed a significant difference between the frequency of the DRB1*03 allele group and the DRB1*15:01 allele (Ribeirão Preto (SP)^25^—DRB1*03: 24.07%-NMO vs 8.62%-MS, *p*^*cF*^ = 0.0254; DRB1*15: 3.7%-NMO vs 37.9%-MS, *p*^*cF*^ = 0.0001 and Rio de Janeiro^33^—DRB1*03:01: 20%-NMO vs 6.4%-MS, *p*^*cF*^ ≤ 0.001; DRB1*15:01: 2.3%-NMO vs 15.4%-MS, *p*^*cF*^ ≤ 0.001). Two other studies showed significant differences only in the distribution of the DRB1*15 allele group (French West indies^26^—8.3%-NMO vs 24.8%-MS, *p*^*F*^ = 0.015; India^31^—9.0%-NMO vs 21.0%-MS, *p*^*cF*^ = 0.001).

## Discussion

The scientific evidence brought by the medical literature in this systematic review confirms that NMO is an associated HLA disease, thus classified because it occurs more frequently in individuals expressing certain *DRB1* or *DPB1* alleles. Evidence of the relationship between the HLA system and the genetic susceptibility has led to numerous studies concerning autoimmune etiopathogenesis.

The HLA complex maps to the short arm of chromosome 6 and provides instructions for making a group of related proteins known as HLA antigens. The human MHC is divided into three regions. The class I region contains the classical *HLA-A*, *HLA-B*, and *HLA-C* genes that encode the heavy chains of these class I molecules, expressed on the surface of most nucleated cells. The class II region contains *HLA-DR*, *HLA-DQ*, and *HLA-DP* genes, each encoding groups of antigens whose expression is limited to antigen-presenting cells (APC): B-lymphocytes, dendritic cells, monocytes, macrophages, endothelial cells, and activated T-lymphocytes. Class I molecules identify cells that are changed, bind to endogenous antigens in the target cells, and present the processed peptides from these antigens to CD8+ T cells (cytotoxic/suppressive), so the changed target cells can be killed by these lymphocytes. Class II molecules on the APC bind to extracellular exogenous proteins, and process and present them to CD4+ T lymphocytes (helper/inducer), initiating an immune response. The Class III region contains loci responsible for 21-hydroxylase, complement components, hormones, MIC molecules, and other signaling molecules such as tumor necrosis factors (TNFs) and heat shock proteins, and is not considered a part of the HLA complex. Nevertheless, it is located within the HLA region, and subject to similar genetic control mechanisms to the HLA genes^37^. As most of the genes located in the MHC complex encode molecules that have a high polymorphism, but low frequency of recombination, the allelic variation between them can make them good markers associated with either protection or susceptibility^17^.

The discovery of the association between *HLA* allelic variants and susceptibility to MS was brought by studies conducted in Denmark in the 1970s^38^. It has been suggested that individuals could develop MS if they inherited certain *HLA* alleles that would make them vulnerable to environmental stimuli, initiating a chain of immunological events that would attack the myelin sheath. More than 500 studies worldwide using genotyping techniques confirmed a strong association of MS with the DRB1*15:01, DQA1*01:02 and DQB1*06:02 haplotype^17,39^.

This systematic review analyzed 13 case–control studies published from 2009 to 2020 that investigated the HLA association with NMO in populations with different ethnic background. Genotyping, with low, medium, or high resolution, was the method used in all eligible studies in the laboratory investigation of HLA class I alleles (*A* and *B*) and HLA class II alleles (*DRB1*, *DQA1*, *DQB1*, and *DPB1*). Only alleles of the *DRB1* locus were genotyped in all the studies. All the studies genotyped alleles of the *DRB1* locus ranging from seven to 30, the number of alleles investigated (Table 2).

The DRB1*03/*03:01 were the most frequently found allelic group and allele, respectively in NMO groups with marked differences according to the ethnic background (Table 3). The allelic frequency (2n) of the DRB1*03 or its subtype *03:01 varied from 10 to 26.2% and the phenotypic frequency (n) varied from 2 to 51.2%. The allelic frequency in Western populations was 14% in Mexican Mestizos^34^, 16.7% in South Brazil^35^, 20.0% in Rio de Janeiro^33^, 20.4% in Spanish Caucasian^27^, 22.0% in French Caucasians^24^, 24% in Danish Caucasians^29^, 24.1% in mulattos from São Paulo^25^ to 26.2% in Afro Caribbean from West French Islands^26^. The phenotypic frequency (n) was 41.5% in Rio de Janeiro^33^, 47.6% in Afro Caribbean^26^ and 51.2% in Netherlands^36^, what means that in Dutch, most NMO patients carried alleles of the DRB1*03 allele group.

The lowest frequencies of the HLA-DRB1*03/ HLA-DRB1*03:01 allele, was found in Asian populations. In Muslim Arabs from Israel^32^, the allelic frequency (2n) was 10% (like NMO and HC), and in India^31^, it was 11%. The phenotypic frequency (n) was 2% in South Japan^30^ (2.6%) and 23% in China^28^ (23.3%).

To compare the DRB1*03 allele group’s association, we used a meta-analysis based on the *OR* and the confidence interval (95% CI) described in the thirteen studies. The general evaluation showed heterogeneity of the *OR* among the studies of only 3.3% (I^2^ = 3.28%; *p* = 0.41). The forest plot (Fig. 2) shows the summary measure of *OR* equal to 2.46 (95% CI 2.01–3.01). That is, patients with NMO are 2.46 times more likely to have the DRB1*03 allele group than controls. In the West, studies are not heterogeneous (I^2^ = 0.00%; *p* = 0.92), with the measure of *OR* equal to 2.38 (95% CI 1.90–2.97), but in Asia the result of the meta-analysis showed a heterogeneity of 67% (I^2^ = 66.91%; *p* = 0.02).

The results of the case–control studies comparing the allelic frequency of the DRB1*03 allele group in NMO with local controls also varied according to the ethnic background. In Caucasian populations, the association of DRB1*03 allelic group in NMO, firstly described in French Caucasians^24^, was only confirmed in the Netherlands^36^ (NMO-51.2% vs controls-27.6%, *p*^*cS*^ = 0.02). In Spain^27^, Denmark^29^, and in the southern region of Brazil^35^ (where 80% of the participants are of European ancestry), such association has not been demonstrated.

However, in Latin American populations, with a high admixed genetic background, DRB1*03 allelic group was associated with NMO in Afro Caribbean^26^ (NMO-26.2% vs controls-13%, *p*^*cB*^ = 0.045), in Mulattoes from Ribeirão Preto^25^ (NMO—24.1% vs controls 8.9%, *p*^*cF*^ = 0.0401), and in Mestizos of Mexico^34^ (NMO vs 14% vs controls 5%, *p* = 0.03). Furthermore, in Rio de Janeiro^33^, where 70% of the population are Afro descendants, the DRB1*03:01 allele was associated with NMO (NMO—41.5% vs controls 22.2%, *p*^*cF*^ = 0.007).

In Asia, despite the low frequency of DRB1*03 allele group, an association with NMO was confirmed in India^31^ (NMO 11% vs controls 2%, *p* = 0.00009). However, no association with this allele and NMO was founding in either Muslin Arabs from Israel^32^ or in patients from Japan^30^ or China^28^.

The strongest association with NMO in Asians was identified with the DPB1*05:01 allele in China^28^ (NMO—90.0% vs controls—55.61%, *p*^*cB*^ = 0.018) and in Japan^30^ (NMO—85.7% vs controls—65.4%, *p* = 0.0074) confirming initial studies in Japanese patients with OSMS^21,22^. No association was found in Caucasians from Western Countries (France^24^ and South Brazil^35^) with DPB1*05:01 allele and NMO. There are no published data on the association of alleles of the *DP* locus in NMO patients with African ancestry.

The ethnicity can influence genetic susceptibility. The frequencies of DPB1*05:01 allele are higher in Asians (44.9–73.1%) than in Caucasians (2.6–5.3%)^22^. In two western populations, France^24^ and the southern region of Brazil with strong European ancestry^35^, the frequency of class II alleles of the *DP* locus in NMO patients and in local controls was also investigated; however, the association of the DPB1*05:01 allele with NMO was not found. These results might be due to the so low frequency of the DPB1*05:01, limiting the statistical power to detect the association^28^.

The association of the DRB1*03 allele group in NMO stratified according to the NMO-IgG status (positive or negative) was also investigated in five studies (Table 3). In French Caucasians^24^, the DRB1*03 allelic group was associated only with the NMO IgG-positive subgroup. A combined analysis in cases from Spain and France^27^ (NMO-AQP4 positive—25% vs controls—10.81%, OR = 2.74, CI 1.58–4.77, *p*^*cB*^ ≤ 0.0008) confirmed the French results. In India^31^, the DRB1*03 allele group’s association only persisted after stratification for AQP4 positivity. However, in Rio de Janeiro^33^, the DRB1*03:01 allele was associated with NMO regardless of the NMO-IgG status. Identification of the NMO-IgG antibody represented a milestone in the knowledge of NMO and related diseases. However, the detection of this antibody showed to be variable according to the population and the laboratory method used. The frequency of NMO-IgG ranged from 44.8 to 72.7% in the studies reviewed here. In the absence of a biological marker, the subgroup NMO IgG-negative may unduly include cases of classic MS, cases of spinal optic MS and cases of MOGAD, so the results on genetic susceptibility in these series need to be interpreted carefully.

The second goal was to verify possible differences between the genetic susceptibility of NMO and other immune-mediated diseases of the CNS.

Eight studies selected for this review, while focusing primarily on the HLA association with NMO, also looked at MS’s genetic susceptibility (data shown in Table 5). The strongest association of the DRB1*15 allelic group with MS worldwide was confirmed in six of the eight studies (Caucasians from France^24^ and Denmark^29^, Latin Americans from the Caribbean^26^, Brazil-SP^25^ and Brazil-RJ^33^ and Asians from India^31^). The DRB1*15 allelic group was not associated with MS in Spanish Caucasians^27^ and Asians from South China^28^.

Differences in the frequency of the DRB1*15 allelic group (MS) and the DRB1*03 allelic group (associated with NMO) were investigated in these eight populations, as illustrated in Fig. 3. In Non-Caucasian populations from Caribbean Islands^26^ and India^31^, a significant difference was found in the frequency of the HLA DRB1*15:01 but not in the frequency of the DRB1*03. Only in two populations living in the Southeast region of Brazil^35^, with strong African ancestry, it was shown that the distribution of both *HLA DRB1* allele group (DRB1*03 and DRB1*15) in NMO is different from that observed in MS. Caucasians do not differ in the frequency of those associated alleles in the groups NMO and MS. As shown in Fig. 3, the DRB1*15 allele group in Denmark^29^, was practically similar in NMO and MS (30% and 35% respectively).

Only one study in the Dutch population with European ancestry^36^ investigated HLA class I and class II alleles in NMOSD and MOGAD diseases. The susceptibility for NMO was strongly associated with the HLA-A*01, B*08, and DRB1*03 but no significant HLA association was found in MOG-IgG–seropositive patients.

Lincoln et al.^40^ investigating the epistatic effect between the *DQA1*, *DRB1* and *DQB1* alleles and their association with MS drew attention to the possibility that the HLA-associated diseases are more haplotypical than allelic. *DR*/*DQ* haplotypes in NMO, MS, and controls were only investigated in the population of Rio de Janeiro^33^ (data shown in Table 6). Among 29 haplotypes, eight were associated with either NMO or MS. The DRB1*03:01-DQA1*05:01/3/5–DQB1*02:01 was the most frequent haplotype (20%) associated with NMO. The haplotype DRB1*15:01–DQA1*01:02-DQB1*06:02 was associated with MS. Therefore, the significant difference in the NMO and MS groups confirmed haplotypic differences in the genetic susceptibility.

**Table 6:** Haplotypes identified in association with NMO and MS, and comparison of NMO and MS groups.

Haplotypes			Allelic frequency (2n) %			NMO vs controls			MS vs controls			NMO vs MS		
*DRB1*	*DQA1*	*DQB1*	NMO	Control	MS	*OR*	CI	*p*^*cF*^	*OR*	CI	*p*^*cF*^	*OR*	CI	*p*^*cF*^
*01:02	*01:01	*05:01	3.1	0	0.5	1.03	1.001–1.06	0.02	1.005	0.995–1.02	0.49	5.94	0.66–53.7	0.16
*03:01	*05:01/3/5	*02:01	20	11	6.4	2.02	1.09–3.75	0.02	0.55	0.27–1.15	0.11	3.67	1.78–7.58	0.0
*04:01	*03:01	*03:02	2.3	4	8.5	0.57	0.15–2.18	0.54	2.23	0.93–5.35	0.07	0.25	0.07–0.89	0.02
*10:01	*01:04/5	*05:01	5.4	1	0.5	5.63	1.15–27.6	0.03	0.53	0.05–5.89	1.00	10.64	1.29–87.6	0.009
*11:01	*05:01/3/5	*03:01	4.6	10	4.3	0.44	0.17–1.12	0.08	0.40	0.17–0.93	0.03	1.09	0.37–3.22	0.88
*13:02	*01:02	*06:04	1.5	4	0.5	0.38	0.08–1.79	0.33	0.13	0.02–1.04	0.04	2.92	0.26–32.6	0.57
*13:03	*05:01/3/5	*03:01	1.5	0.5	3.7	3.11	0.28–34.6	0.56	7.696	0.94–63.2	0.03	0.40	0.08–1.98	0.32
*15:01	*01:02	*06:02	2.3	4	15	0.57	0.15–2.18	0.54	4.38	1.95–9.84	0.00	0.13	0.04–0.44	0.0

MS, Multiple Sclerosis; NMO, Neuromyelitis Optica; *OR*, *Odds Ratio*; CI, Confidence Interval; *p*^*cF*^, *p* corrected by Fisher’s exact test.

Adapted from Alvarenga et al. (Table 2)^33^.

Genetic interactions of the DRB1*03:01–DQA1*05:01/3/5–DQB1*02:01 haplotype and *DRB1* alleles have been described in systemic autoimmune diseases and in organ-specific immune-mediated diseases with the involvement of autoantibodies against extra and intracellular antigens. Some of these diseases occur more frequently in patients with NMO than in the general population^41^.

Data from five series of NMO patients here reviewed corroborate these data. Other autoimmune diseases occurred in 6.1%^33^, 14.6%^29^, 18.2%^27^ 26.7^35^–33.3%^24^. Overall, 23 autoimmune diseases were identified, the most frequent being Hashimoto`s thyroiditis (n = 7), Sjögren Syndrome (SS) (n = 4), Diabetes Mellitus Type 1 (T1DM) (n = 3), myasthenia gravis (n = 2), rheumatoid arthritis (RA) (n = 2), anti-phospholipid antibody syndrome (n = 2), ulcerative colitis (n = 1), celiac disease (n = 1) and Systemic Lupus Erythematosus (SLE) (n = 1). Two cases of cancer in association with NMO were also described (lung and breast).

Genetic factors have been suggested to explain the association between systemic autoimmune diseases and NMO. One possibility would be the *HLA* genes related to humoral immunity are involved in the regulation of autoimmune functions in those immune-mediated diseases. The DRB1*03:01–DQA1*05:01/3/5–DQB1*02:01 haplotype is associated with T1DM^42^, SLE, SS^43,44^. The DRB1*03 allele group is associated with SLE, Autoimmune Polyglandular Syndrome, and Graves' Disease^45–47^. The DQB1*04:02 allele is associated with primary biliary cirrhosis, with T1DM and juvenile idiopathic arthritis^48–50^. Lichen planus, RA, and ovarian cancer are associated with DRB1*10 and invasive squamous cell cancer of the cervix with DRB1*10:01^34^. The DRB1*04:05 allele has also been associated with other autoimmune diseases in the Asian population^51,52^. Although less frequent, research has linked autoimmune diseases to HLA class I, such as T1DM, primary SS, and, more often, optic neuritis^53–55^. As shown in this review, the DRB1*03 allelic group was associated with NMO in different populations.

Other shreds of evidence link NMO with other autoimmune diseases. Acute events of optic neuritis and transverse myelitis in SLE and SS’s raised the following question: would they occur due to a genetic influence on the autoimmunity shared between these diseases? Would they be complications of rheumatic diseases affecting the CNS^41^? In SLE, inflammation damages the lungs, kidneys and CNS membranes, which express the AQP4 protein. Autoantibodies typically associated with SLE bind to DNA and RNA proteins, ribosomal proteins, and phospholipids. NMO-IgG antibodies have been detected in the serum of patients with SS or SLE and concomitant NMOSD, but not in the serum of patients with SLE or SS who do not have NMO spectrum diseases^56^. Based on these data, Pittock et al.^57^ suggested that the occurrence of SLE/SS or autoantibodies in association with diseases of the NMO spectrum combined with seropositivity for the NMO-IgG antibody indicates that there is an association of these diseases. For this reason, they were included among the NMO spectrum syndromes^11^.

One GWAS study analyzing exclusively NMO genetic risk factors in Caucasians showed an association with the DRB1*03:01–DQA1*05:01/3/5–DQB1*02:01 haplotype and the class I, HLA-B*08:01 and HLA-C*07:01alleles in NMO subgroup positive for the NMO-IgG. Additionally, a reduced copy number variation (CNV) in the region of complement component *C4* encoded in the MHC class III region was found. Estrada et al.^58^ suggested that the *C4* deletions could be the functional driver of the NMO association and call the attention that the same *C4* CNV and DRB1*03:01–DQA1*05:01/3/5–DQB1*02:01 haplotype were risk factors for SLE.

### Limitations

We have identified some limitations in these studies, such as the low number of NMO cases analyzed in each study (ten studies with 45 or fewer NMO patients). This is explained by the fact that NMO is a rare disease and only recognized as a different condition from MS by specific diagnostic criteria after 1999. Furthermore, another limitation was the low resolution of the genotyping technique since it was limited, in most studies, only to the typing of HLA-DRB1*03 allele group, without specification of its subtypes; as well as the small number of studies genotyping *DR/DQ* alleles to identify the haplotypes associated with NMO.

Finally, the genetic susceptibility of the NMO group negative for AQP4-Ab needs to be analyzed with caution because optic spinal disease could be related to Asian type MS, Conventional Multiple Sclerosis, or MOG-IgG related disorders.

## Conclusions

NMO is an HLA associated disease.

Patients with NMO are 2.46 times more likely to have the DRB1*03 allelic group than controls.

Alleles of the DRB1*03 group, specifically the DRB1*03:01, conferred genetic susceptibility to NMO in most of Latin Americans, in half of the Caucasians and in one-quarter of the Asians. In Far East Asian, the genetic susceptibility for NMO is associated with the DPB1*05:01 allele.

Most of the studies confirmed the DRB1*03 allele group’s association with NMO positive for the NMO-IgG antibody.

The genetic susceptibility for NMO differed from MS in Latin America populations with a high ethnic African background.

In the Netherlands, the DRB1*03:01 allele was associated with NMO, but no HLA association was found with MOGAD. Those findings bring new evidence that NMO, MS and MOGAD are different immune-mediated CNS conditions.

It is recommended that new studies with a greater number of patients analyzed by the four-digit *HLA DR/DQ* alleles immunophenotyping technique be performed in different populations to increase knowledge about genetic susceptibility in NMO.

## Methods

### Selection of the articles

A systematic review of the literature was carried out by a search in the MEDLINE (Medical Literature Analysis and Retrieval System Online) via PubMeb’s updated version interface, LILACS (Scientific and Technical Literature of Latin America and the Caribbean) via VHL (Virtual Health Library) and SciELO (Scientific Electronic Library Online) electronic databases. The search for publications in any of the three languages, English, Spanish or Portuguese was done by two independent evaluators (LFC and HAF). The period for inclusion was 2009 to March 31, 2020. The search strategy used the combined MeSH terms “Neuromyelitis Optica” and “HLA antigens”, and the combined text words Neuromyelitis Optica and HLA association studies.

Studies considered for this review: case–control studies (association studies) analyzing genetic susceptibility through genotyping of HLA genes in human subjects with NMO according to international diagnostic criteria^5,10,14^ and only publications in English, Spanish, or Portuguese languages. Case reports, reviews, publications not related to the review's objectives, and publications in other languages were excluded. Articles identified in more than one database were considered only once. The papers which fulfilled the eligibility criteria were included in the qualitative and quantitative analyzes.

### Outcomes

The primary outcome was the association of the HLA alleles with NMO. Secondary outcomes were a comparison of the genetic susceptibility in NMO and MS.

### Study quality evaluation

The selected articles were submitted to the STROBE evaluation method (Strengthening the Notification of Observational Studies in Epidemiology), for case–control studies^59^. Two evaluators (LFC and HAF) addressed the questions, with a maximum score of 22, equivalent to the number of items presented in the STROBE instrument. We regarded studies that scored “15–22” as high quality, those that scored “7–14” as moderate quality, and those that scored “0–7” as low quality (Supplementary Table S1).

This review employed the guidelines indicated in the MOOSE (Meta-analysis Of Observational Studies in Epidemiology) and PRISMA (Preferred Reporting Items for Systematic reviews and meta-analyses) Consensus Statements^60,61^.

### Statistical analysis

Several comparisons were noted in the included studies; NMO vs controls, NMO vs controls stratified by the NMO-IgG/AQP4-IgG status; NMO vs MS and NMO vs MOGAD. The statistical analysis applied allelic frequencies expressing number of alleles (2n) or phenotypic frequencies (n) indicating the number of participants carrying specific allele. Frequencies of HLA alleles were compared using the chi-square test (*p*) and corrected by Fisher´s exact test (*p*^*cF*^), Bonferroni (*p*^*cB*^), or Sidak (*p*^*cS*^) methods. The level of significance was < 0.05. *OR* with 95% confidence interval (CI) was calculated for each comparison.

A meta-analysis by mixed-effects models was performed using the metaphor library (2010)^62^ of software R version 3.3.2 (2016). To evaluate the studies' heterogeneity, the I^2^ statistics of Higgins and Green^63^ were used. The forest plot chart was used to present the results.

## Supplementary Information

Supplementary Information.
