# Supplementary material for: Neuromyelitis optica is an HLA associated disease different from Multiple Sclerosis: a systematic review with meta-analysis
Source: Sci Rep. 2021 Jan 8;11:152. doi: 10.1038/s41598-020-80535-3 (PMC7794341; doi:10.1038/s41598-020-80535-3)
Supplement: Supplementary file 1 — Supplementary Information. [file 41598_2020_80535_MOESM1_ESM.pdf]

Neuromyelitis optica is an HLA associated disease different from Multiple Sclerosis. A systematic review with meta-analysis.

Marcos Papais Alvarenga[1,2,3], Luciana Ferreira do Carmo[1], Claudia Cristina Ferreira Vasconcelos[1], Marina Papais Alvarenga[1], Helcio Alvarenga Filho[1,3], Cleonice Alves de Melo Bento[1], Carmen Lucia Antão Paiva[1], Laura Leyva-Fernández[4,5], Óscar Fernández[6], Regina Maria Papais Alvarenga[1,2]*

[1]Programa de Pós-Graduação em Neurologia, Universidade Federal do Estado do Rio de Janeiro (UNIRIO), Rua Mariz e Barros 775, Rio de Janeiro/RJ 20270-004, Brazil.

[2]Departamento de Neurologia, Hospital Federal da Lagoa, Rua Jardim Botânico 501, Rio de Janeiro/RJ 22470-050, Brazil.

[3]Universidade Estácio de Sá (UNESA), Avenida Ayrton Senna, 2800, Barra da Tijuca, Rio de Janeiro/RJ 22775-003, Brazil.

[4]Instituto de Investigación Biomédica de Málaga-IBIMA, UGCNeurociencias, Hospital Regional Universitario de Málaga, Avenida de Carlos Haya sn, Málaga 29010, Spain.

[5]Red Temática de Investigación Cooperativa: Red Española de Esclerosis Multiple REEM (RD 16/0015/0010).

[6]Instituto de Investigación Biomédica de Málaga-IBIMA, Hospital Regional Universitario de Málaga, Avenida de Carlos Haya sn, Málaga 29010, Spain.

***Correspondence to:**

Regina Maria Papais Alvarenga, Programa de Pós-Graduação em Neurologia, Universidade Federal do Estado do Rio de Janeiro (UNIRIO), Rua Mariz e Barros 775, Rio de Janeiro/RJ 20270-004, Brazil. Tel/Fax: 55 21 22642123.

e-mail: regina_alvarenga@hotmail.com

Supplementary Table S1 | STROBE assesment

Studies	(S1+S2)/2	Score
Zéphir, H. et al[24]	18	HQ
Brum, D.G. et al[25]	17	HQ
Deschamps, R. et al[26]	17	HQ
Blanco, Y. et al[27]	14	MQ
Wang, H. et al[28]	15	HQ
Asgari, N. et al[29]	17	HQ
Yoshimura, S. et al[30]	16	HQ
Pandit, L. et al[31]	12	MQ
Brill, L. et al[32]	17	HQ
Alvarenga, M.P. et al[33]	18	HQ
Alonso, V.R. et al[34]	11	MQ
Kay, C.S. et al[35]	16	HQ
Bruijstens, A.L. et al[36]	19	HQ

STROBE[59]: Strengthening the Reporting of Observational Studies in Epidemiology; S1: researcher 1/STROBE 1; S2: researcher 2/STROBE 2. Score “15–22″: high quality (HQ); “7–14″: moderate quality (MQ); “0–7″: low quality (LQ).

The selected articles were submitted to the STROBE evaluation method (Strengthening the Notification of Observational Studies in Epidemiology), for case-control studies[59]. The questions were addressed by two evaluators (LFC and HAF), with a maximum score of 22, equivalent to the number of items presented in the STROBE instrument. We considered studies that rated “15–22″ as high quality, those that rated “7–14”as moderate quality and those that rated “0–7″ as low quality. The grades attributed by the evaluators for each of the studies had the average calculated to determine the score. Of the thirteen studies evaluated, ten were classified as high quality and three were classified as medium quality (Supplementary Table S1).
